# Interaction of polyethylene glycol with cytochrome c investigated via in vitro and in silico approaches

**DOI:** 10.1038/s41598-021-85792-4

**Published:** 2021-03-19

**Authors:** Zahoor Ahmad Parray, Faizan Ahmad, Mohamed F. Alajmi, Afzal Hussain, Md. Imtaiyaz Hassan, Asimul Islam

**Affiliations:** 1 Centre for Interdisciplinary Research in Basic Sciences, Jamia Millia Islamia, Jamia Nagar, New Delhi, 110025 India; 2 Department of Pharmacognosy College of Pharmacy, King Saud University, Riyadh, Saudi Arabia

**Keywords:** Biochemistry, Biophysics, Biotechnology, Cancer, Structural biology, Diseases

## Abstract

One of the significant proteins that have attracted research groups due to virtue of being a potent selective anticancer drug target and property of triggering apoptosis upon release in cytoplasm is cytochrome *c* (cyt *c*). The mechanical transformations due to the macromolecular crowding in membrane in the mammalian cell are proposed to be useful inductors of changes in volume. It is very interesting to know that mitochondrial function were observed to be improved by polyethylene glycol (PEG) interaction, which in turn inhibits the cyt *c* (*a* pro-apoptotic cell death factor). In this work, the effect of polyethylene glycol of molecular weight 4 kilo Dalton (PEG 4 kDa) was investigated to highlight the structural transformations (tertiary and secondary structure) in cyt *c* using a choice of spectroscopic techniques (including UV–Vis absorption, near-UV, far-UV and Soret circular dichroism and fluorescence spectroscopy), which shows noteworthy shifts in the secondary and tertiary structures at higher concentrations of PEG 4 kDa with small changes in the heme-globular interactions. The size distribution changes of native protein treated with various concentrations of the crowder were observed and analyzed by dynamic light scattering (DLS). The interaction studies of the crowder with the protein was observed and analyzed by FTIR, isothermal titration calorimetry, time resolved fluorescence and molecular docking. The investigations suggested that the structural changes in the protein occurred due to soft interactions of PEG 4 kDa, which usually destabilizes proteins. The experimental evidence in this study proposed that crowding could be another approach to mechanical super-competition and free of certain markers that could aid in the identification and control of various diseases. This study suggests that crowders at specific concentrations, which softly interact with proteins, can be exploited as remedy for various diseases.

## Introduction

The PEG–protein interactions are feasible because of change in crowders hydrophilicity into amphiphilicity with increasing molecular weight of PEG (MW_PEG_)^1^. Earlier it was assumed that no or very weak interaction occurs between PEG and proteins, however, the previously reported studies^1–10^ gave new evidences that protein and PEG in aqueous solution holds various types of interactions. Such interactions are important not only for understanding the structural effects and changes in activity of proteins in solutions but also reliable to design materials for specific purposes^4,5,11–18^. PEGs are broadly used in a range of pharmaceutical formulations, including topical, parenteral, oral, ophthalmic and rectal preparations^1,17^. PEGs induce intermediate states (molten and pre-molten globules) in heme-proteins^2,4,5,10^. On introducing to patients, PEGs tend to increase blood clot tendency and causes clumping of cells and death occurrence from embolism if given rapidly^19^. Few evidences have suggested that PEG is a greatly efficient as chemo-preventive mediator against colon cancer; conversely, the process and mechanism(s) remains fundamentally unfamiliar^20,21^. The researchers had observed that the antigenic site, II of G protein of Rabies virus (RV) was blocked by PEG 4 kDa. PEG 4 kDa was found to be docked successfully on the binding pocket encompassing antigenic site III of G protein of Chandipura virus (CHPV) instead of expected antigenic site II as in RV^9^, where it was observed that PEG 4 kDa may have neutralizing effect^9^.

Also, various investigational evidences showed that crowding-induced death may possibly a choice of model for super-competition or more specifically for automatic super-competition, free of recognized markers, which endorse tumor growth^22–24^. For better modification at the single-cell phase of proliferation pace, cell volume and death of cell needs tissue size regulation^25^. The structural modifications in the cell plasma membrane and its macromolecular crowding are believed to be useful inductors that change the mammalian cell size and volume. Conversely, the character of the cellular sensors specifically for osmotic alterations left unfamiliar^26^. PEG acts as a membrane repair agent (instantly repairs neuronal membrane disruptions) and oxidative injuries were reduced^8^. In additional studies, other researchers showed that PEG markedly reduces the caspase 3 activity, hence apoptotic cell death^8,20^. They suggested that PEG on interaction with mitochondria (segregated) increases its function and inhibits the cytochrome *c* (a pro-apoptotic cell death factor) release in cytoplasm^8^. Moreover, it is known that PEG’s are crystallizing agents^11^, precautions should be taken whilst using PEG’s as crystallizers because PEG is also a destabilizing agent at higher concentrations^4,5,16,27,28^. It should be noted that most of the PEGs and other crowders on interaction disrupts heme in the proteins (cytochrome *c* and myoglobin)^4,5,10,29–31^. Moreover, the effect of PEG 4 kDa at small concentrations on cyt *c* has been reported where it was observed that PEG 4 kDa increases the auto-oxidation of cyt *c*, however changes are insignificant^31^. In addition, the study proposed a model showing that heme is dislocated due to dehydration of some surface exposed hydrophobic residues i.e. Ile81 and Val83; however their spectroscopic techniques showed no significant changes^31^ and are comparable to our results at lower concentrations. They have used small concentrations of PEG 4 kDa, however we are dealing with macromolecular crowding and its effect on the protein we have used variable concentrations (low to high) of the same molecular weight of PEG in similar conditions but through different approaches.

From the above studies so far it is confirmed that PEGs showed interaction with various proteins including cyt *c.* In this study various concentrations from low to high (50–300 mg ml^−1^) of PEG 4 kDa were taken to observe effects of crowding on the structure of cyt *c* using various spectroscopic techniques. Moreover, the various techniques were exploited to investigate the nature of binding of the crowder with the protein.

## Materials and methods

### Materials

Commercially available lyophilized forms of horse heart cytochrome *c* and 8-anilino-1-napthalene sulphonic acid (ANS) were procured from Sigma chemical company (USA). Hydrochloric acid (HCl), PEG 4 kDa and sodium hydroxide (NaOH) were procured from Merck (India). Sodium phosphate monobasic and di-sodium hydrogen phosphate anhydrous were purchased from Himedia (Germany). The filters of pore size equal to 0.22 μm were purchased from Merck Millipore Ltd. (Cork).

### Methods

#### Preparation of protein and reagents^10,32^

Lyophilized powdered cyt *c* (75 mg ml^−1^) was dissolved in required amount of 50 mM phosphate buffer, followed by oxidation using potassium ferricyanide^33^. The prepared solution of the protein was then dialysed against 50 mM phosphate buffer solution at pH 7.0 and 4 °C with several changes, and it was observed that 2 L of the buffer were adequate to take out potassium ferricyanide and other salts in excess. After dialysis, the protein was filtered using a 0.22 μm Millipore filter. The protein concentration was determined by Beer-Lamberts law (*c* = *A*_410_/*εl*), where c is concentration of protein in molar, *l* is the path length of cell in cm, *A* is the absorbance value at wavelength of 410 nm and *ε* is the molar absorption coefficient at 410 nm (*ε*_410_, M^−1^ cm^−1^), the value estimated was 106,100 M^−1^ cm^−1^ close to the reported one^34^ and that for ANS at 350 nm was equal to 5000 M^−1^ cm^−1^^35^.

The requisite amount of the crowder molecule (PEG 4 kDa) and denaturant (guanidinium chloride, GdmCl) were dissolved in the phosphate buffer followed by filtration using the Whatman filter paper No. 1, and all the concentrations of the stock solutions were observed from values obtained from refractive index^36,37^. The required solutions were prepared in degassed buffers to carry out optical measurements. Each sample of different concentration of PEG 4 kDa were prepared in triplicate at 25 ± 1 °C and incubated for overnight.

### Spectroscopic techniques

#### UV–visible spectra measurements^10,32^

Spectral measurements were made by Jasco V-660 UV–vis spectrophotometer connected to a Peltier type temperature controller (ETCS761), which controls temperature. The protein of 6–7 µM was taken for all measurements of absorbance in the region of 240–700 nm (near-UV and Soret- absorption) using 1.0 cm cuvette path length.

#### Circular dichroism (CD) studies

The spectral measurements of circular dichroism (CD) was carried out by Jasco Spectropolarimeter (J-1500 model), fixed with a circulation bath (MCB-100). For the experimental measurements of CD spectra (near- and far-UV), the protein concentration was taken as 14–16 µM in 1.0 and 0.1 cm path length cuvette correspondingly. Soret CD spectra measurements were done in the range of 370–450 nm in 1.0 cm path length cuvette. The experimental equipment exploited was standardized consistently using D-10 camphor sulphonic acid. Various accumulations were set for each sample together with baseline for improvement of better signal to noise ratio. 4–5 L min^−1^ of nitrogen was flushed constantly to lessen level of noise. The CD (mdeg) records were converted to parameter which is concentration independent i.e., [*θ*]_λ_ (deg cm^2^ dmol^−1^), the mean residue ellipiticity (MRE), via following equation:

1 $$\left[ \theta \right]_{\lambda } = {\text{M}}_{0} \theta_{\lambda } /10lc$$

where *θ*_λ_ is ellipticity at wavelength λ, in millidegrees, *M*_0_ is the mean residue weight of the protein, *c* is the protein concentration used in gm cm^−3^, and *l* is path length of cuvette in centimeters. [*θ*]_222_ and [*θ*]_208_ probes were exploited to monitor changes in secondary structure of the protein and α-helical content estimation in the absence and presence of the crowder^38,39^.

#### Fluorescence spectra measurements^10,32^

Jasco FP-6200/STR-312 spectro-fluorimeter was exploited for the fluorescence measurements. The emission and excitation slits were fixed at 10 nm and cuvette of 1.0 cm of path length was applied in the experiment. The external thermo-stated water bath connected to spectro-fluorimeter controls cell temperature. The excitation wavelength used for measurements was 280 nm and 300–400 nm of wavelength region considered emission spectra. For ANS measurements 400–600 nm of emission spectra with excitation wavelength of 360 nm was considered.

#### Infrared spectroscopy studies^32^

Measurements of Fourier transform infrared (FT-IR) spectra were carried out with Bruker Tensor 37, which is adaptable grade instrument used in research. The optics of technique and compartment where sample was placed were continuously flushed out with dry nitrogen. 0.5 mg ml^−1^ of the protein (cyt *c*) concentration was taken for each measurement. The experiment was performed without and with PEG 4 kDa and 6 M GdmCl. A drop of sample was placed on the plastic metal frame. In order to observe various structural alterations in cyt *c* exposed to PEG 4 kDa and 6 M GdmCl, spectra were carried out in the wavenumber range of 1150 to 3500 cm^−1^.

#### Size distribution measurements^32,40^

Malvern Zetasizer Nano ZS instrument was used to carry out all size distribution measurements at 25 °C and pH 7.0. The detection angle of 12.8° and scattering angle of 175° plus a Helium–Neon laser having power of 4 mW, at the wavelength of 632.8 nm and with beam diameter size of 0.63 nm (1/e2) was set in all experiments. The samples of cyt *c* in different solvents conditions i.e., buffer, PEG 4 kDa (50 and 300 mg ml^−1^) and 6 M GdmCl were placed in standard Malvern polystyrene cuvettes of 10 mm for size measurements, respectively. The software Zetasizer Ver. 7.13 of Malvern Panalytical was exploited for the data analysis. The measurement of each sample was repeated 3–4 times.

#### Isothermal titration calorimetry (ITC) measurements

Isothermal titration calorimetric experimental measurements were done from VP ITC Calorimeter (Model MicroCal, Northampton, MA). All the experiments were carried out at 25 °C (298 K) in phosphate buffer (pH 7.0) with strength of 50 mM. PEG 4 kDa was titrated into calorimeter cell already filled with cyt *c*, the concentration ratio of the crowder and the protein was 30:1 respectively. PEG 4 kDa was filled in syringe and 10 µl aliquots were injected in every 260 s accept first which was 5 µl. The titration of PEG 4 kDa into cyt *c* gives data points, which were normalized against molar ratio of the crowder and followed by evaluation using Origin software (installed by MicroCal). The data points were best fitted by sequential binding model and generated various thermodynamic parameters such as the stoichiometry (*N*), change in entropy (Δ*S*°), change in the binding enthalpy (Δ*H*°) and the association constant (*K*_a_). From these primary parameters, secondary and significant parameter change in Gibbs free energy (Δ*G*°) was calculated using following equations:

2 $$\Delta G = - RTlnKa = \Delta H - T \Delta S$$

where *T* is the absolute temperature in Kelvin (*K*) and *R* is the gas constant.

#### Time resolved fluorescence measurements^32,40^

Time-resolved fluorescence studies were done at 25 °C via Modular Fluorescence Lifetime System (Delta Flex) connected with DPS-1 detector power supply, external control DD-C1 Pico diode controller (Delta Diode) and DH-HT high throughput TCSPC controller (Delta Hub) of HORIBA Scientific. The excitation and emission wavelengths for these measurements were set at 280 nm and 342 nm, respectively. The fluorescence lifetime data measurements were scanned in the peak to 10,000 counts. The instrumental response function (IRF) was successively obtained using a time calibration of 114 ps/channel and a scattering solution. The analysis of data was created from the sum of exponentials, using a non-linear least square convolution analysis of the inputs using software for analysis by equation:

3 $$f\left( t \right) = \mathop \sum \limits_{i = 1}^{n} {\text{a}}i\exp \left( {\frac{{ - {\text{t}}}}{{{\uptau }i}}} \right)$$

where τ_i_ are the decay times, n is the number of decay times and a_i_ is the comparative contribution of the components at t = 0. The goodness of fit was reviewed in requisites of equally weighted residuals and chi-squared (χ^2^) values. The function time-resolved fluorescence decays were analyzed by the impulse response using software of HORIBA EzTime^41^.

#### Computational studies

PEG 4 kDa was docked to a macromolecule (cyt *c*) by virtual molecular screening through the PyRx software. PyRx software is written in Python programming language with sensitive client’s interface that functions on most of the operating systems (Windows, Linux and Mac OS). The softwares such as AutoDock 4.2, AutoDockVina, Open Babel, Mayavi, Vina and AutoDock 4.2 are used by PyRx as docking tools^42^. The input files ligand (PEG 4 kDa) and macromolecule, Cyt *c* (PDB ID: 1hrc) in .pdb format were changes to .pdbqt files using Autodock software. After preparing the files, it was subjected to docking by means of AutoDock 4.2 and Vina^43^. Grid box dimensions were set to be X, Y and Z conformations equal to 42 Ǻ, 39 Ǻ and 44 Ǻ, respectively. The grid space size was assigned perfectly which allows selecting search space for the protein to execute docking, typically the protein’s binding site. The interaction between cyt *c* and the respective PEG was interpreted using the Lamarckian Genetic Algorithm (LGA). Once the Vina calculations were done the results of binding affinity (kcal mol^−1^) interactions of various conformation ligand binding with macromolecule are provided by software in a table. Finally, the best docked complexes of protein–ligand were further modified and analyzed using visualizer PyMOL and 2D interaction plot was generated by Discovery studio^44^.

## Results

### UV–visible absorption studies

**Figure 1:**
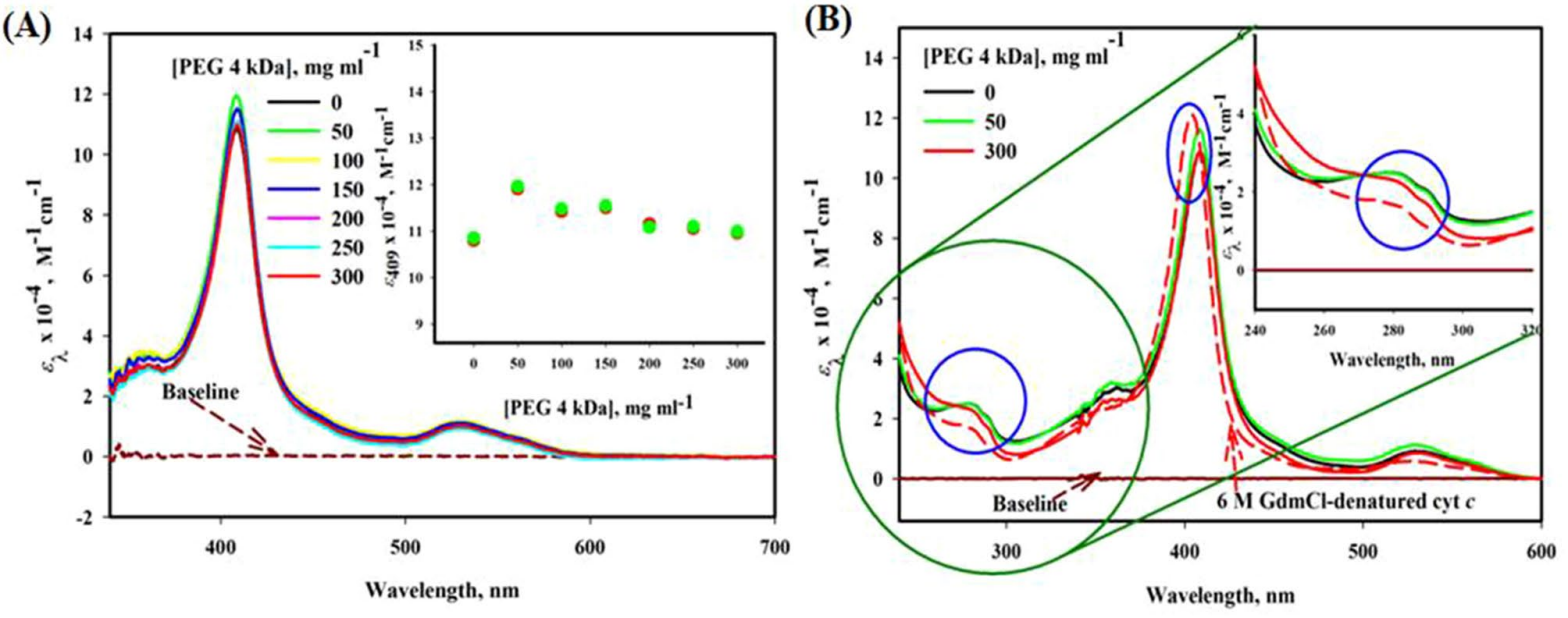
(**A**) Absorption spectra (Soret region) of cyt *c* in the presence of different concentrations (0–300 mg ml^−1^) of PEG 4 kDa at pH 7.0 and 25 °C. Inset shows a plot of *ε*_409_ versus [PEG 4 kDa], where green, red and black circles are data points of triplicate measurements. (**B**) Near-UV and Soret absorption spectra of cyt *c* in the presence of different concentrations (0, 50 and 300 mg ml^−1^) of PEG 4 kDa and 6 M GdmCl at pH 7.0 and 25 °C.

To study the change in structural conformation of proteins and interaction studies, UV–visible spectroscopy is a reliable basic technique of spectroscopy^45^. Figure 1A,B reveals the UV–visible absorbance spectra of cyt *c* without and with PEG 4 kDa, which shows that cyt *c* has several absorption bands in the wavelength (*λ*) regions of 280 nm (arises because of the aromatic side chains of Tyr and Trp residues)^46^, Soret region at 409–410 nm (due to heme) and an oxy-deoxy band (small) around 500–600 nm region^47^. Table S1 provides the significance of these probes, which monitors the change in the environment of aromatic amino acids, heme and various heme-protein residue interactions. The Soret band around 409 nm of cyt *c* in buffer signifies that the protein is in native condition. The figure shows increase in absorbance at lowest concentration and followed by insignificant decrease in absorbance as concentration is increased to 300 mg ml^−1^ of PEG 4 kDa. Red, green and black circles in the inset of this figure are data points of triplicate measurements. Figure 1B extends *λ* in the region of 240 to 600 nm of cyt *c* exposed to 0, 50 and 300 mg ml^−1^ of PEG 4 kDa, showing that the change around 280–300 nm is maximum resulted by highest concentration of PEG 4 kDa whereas lowest shows unaffected (see Inset of Fig. 1B). There was no significant change observed in the wavelength region of oxy-deoxy band (500–600 nm) of cyt *c* due to PEG 4 kDa at all concentrations, in contrast 6 M GdmCl badly affects this region.

### CD spectroscopy measurements

#### Soret-CD

**Figure 2:**
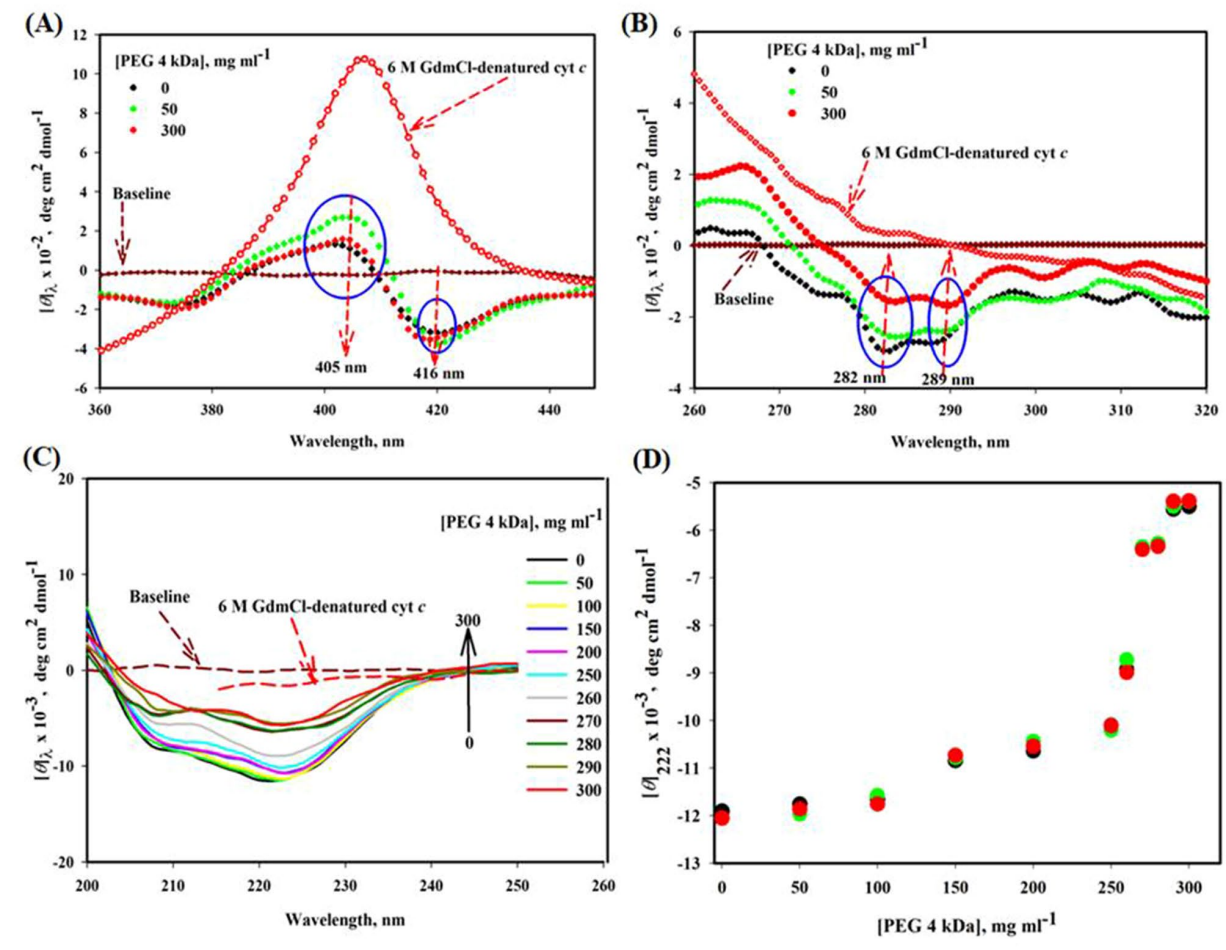
(**A**) Soret-CD, (**B**) near-UV CD and (**C**) far-UV CD spectra of cyt *c* in the presence of different concentrations (0–300 mg ml^−1^) of PEG 4 kDa and 6 M GdmCl at pH 7.0 and 25 °C. (**D**) A plot of CD signal at 222 nm, [***θ***]_222_ versus [PEG 4 kDa].

To confirm the UV–Vis absorption results (Fig. 1A,B), Soret-CD spectra of cyt *c* treated with and without PEG 4 kDa were taken (see Fig. 2A). To observe the changes in heme environment, [*θ*]_405_ and [*θ*]_416_ are excellent probes for observing the interaction strength of Met80 and Phe82 with heme^48–52^. Figure 2A shows Soret CD spectra of cyt *c* treated with 0, 50 and 300 mg ml^−1^ of PEG 4 kDa and 6 M GdmCl. This figure shows that the protein in the presence of 50 mg ml^−1^ of PEG 4 kDa has significant increase in the value of [*θ*]_405_ and almost no change in the value of [*θ*]_416_ of the protein, conversely the protein shows insignificant changes due to higher concentration of PEG 4 kDa i.e., 300 mg ml^−1^. This figure also shows that 6 M GdmCl completely denatures the protein which results in loss of peak around 416 nm.

#### Near-UV CD

To examine the effect of PEG 4 kDa on Trp and Tyr environment and to confirm the results of UV–vis absorption studies, the near-UV CD, which is an excellent probe to check tertiary structural changes^53^, was explored. The earlier reports showed that cyt *c* in buffer have two negative peaks i.e., 282 and 289 nm (see Fig. 2B)^54,55^. In addition, Fig. 2B shows spectra of cyt *c* exposed to 50, 300 mg ml^−1^ of PEG 4 kDa and 6 M GdmCl at pH 7.0 and 25 °C. This figure shows maximum tertiary structure loss of cyt *c* due to 300 mg ml^−1^ PEG 4 kDa and complete loss due to 6 M GdmCl.

#### Far-UV CD

To observe changes in the elements of secondary structure, the far-UV CD technique was explored. At 208 nm and 222 nm, there are two strong CD signals in cyt *c* which define the characteristics of all α-protein^38^. Figure 2C shows far-UV CD spectra of the protein treated with PEG 4 kDa at different concentrations (0–300 mg ml^−1^). The denatured cyt *c* spectrum, exposed to 6 M GdmCl is also given in the figure. A plot of CD signal at 222 nm, [*θ*]_222_ versus [PEG 4 kDa] is also shown in the Fig. 2D, where it is seen that PEG 4 kDa decreases [*θ*]_222_ with increase in the concentration, hence decreases secondary structure. Red, green and black circles in the inset of this figure are data points of triplicate measurements. Table 1 shows the helical content of each state measured at [*θ*]_208_ and [*θ*]_222_. The secondary structural content (α-helical) of the native protein is in conformity to that of reports earlier^56,57^. Table S1 provides the significance of different probes of Soret, far and near-UV CD used to monitor the secondary, tertiary and various heme-globular interactions.

**Table 1:** Secondary structural content of cyt *c* exposed to different concentration of PEG 4 kDa.

[PEG 4 kDa], mg ml^−1^	% α-helix^a^ [*θ*]_222_	% α-helix^b^ [*θ*]_208_
0	43.9 (± 2.5)^#^	41 (± 2.0)^#^
50	43.8 (± 2.0)^#^	39.96 (± 1.6)^#^
300	26.3 (± 1.4)^#^	22.4 (± 1.0)^#^

Values calculated using equations of ^a^Morrisett et al.^39^ and ^b^Greenfield and Fasman^38^; ^#^with each parameter signifies the mean error.

### Fluorescence measurements

#### Intrinsic/Tryptophan fluorescence

**Figure 3:**
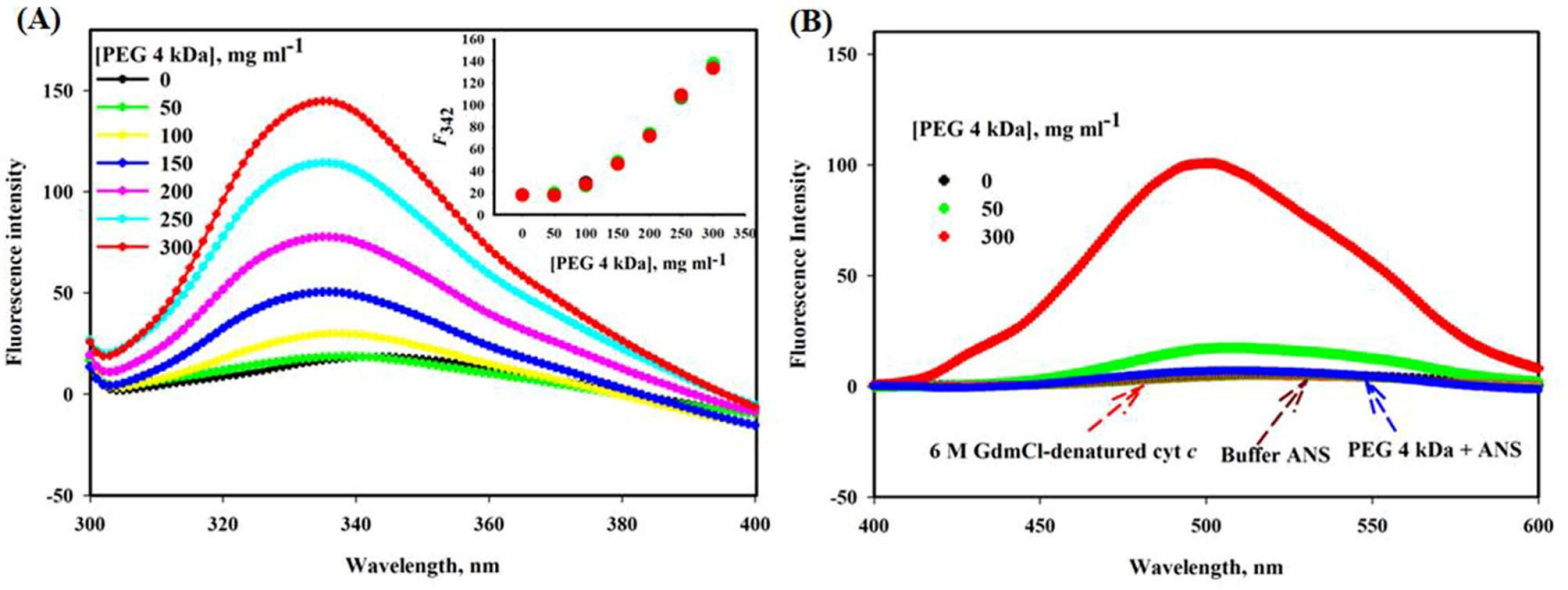
(**A**) Fluorescence spectra of cyt *c* in the presence of different concentrations (0–300 mg ml^−1^) of PEG 4 kDa. Inset of this figure shows a plot of *F*_342_ (fluorescence intensity at 342 nm) versus [PEG 4 kDa], where green, red and black circles are data points of triplicate measurements. (**B**) ANS fluorescence spectra of cyt *c* in the presence of different concentrations (0, 50 and 300 mg ml^−1^) of PEG 4 kDa, buffer + ANS, PEG 4 kDa + ANS and 6 M GdmCl at pH 7.0 and 25 °C.

Figure 3A depicts the intrinsic fluorescence spectra of cyt *c* exposed to various PEG 4 kDa concentrations (0, 50, 100, 150, 200, 250 and 300 mg ml^−1^). Inset of this figure shows the plot of fluorescence intensity at 342 nm, *F*_342_ versus [PEG 4 kDa] in mg ml^−1^, where it is seen PEG 4 kDa increases *F*_342_ with increase in the concentration. Red, green and black circles in the inset of this figure are data points of triplicate measurements.

#### ANS binding Studies

The surface exposed hydrophobic patches on the protein can be probed using ANS fluorescence ^58^. Figure 3B shows ANS spectra of buffer, protein in buffer and protein exposed to 6 M GdmCl, and it is confirmed that neither of the spectra shows binding to ANS hence no increase in fluorescence intensity and/or blue shift. This figure also shows ANS of the protein exposed to 50 and 300 mg ml^−1^ PEG 4 kDa, which shows both blue shift and increase in fluorescence intensity.

Table S1 provides the significance of *F*_342_ and ANS binding (increase in the fluorescence intensity and shift in wavelength) probes to monitor the changes in the tertiary structure and hydrophobic patches on the surface of the protein respectively.

### Fourier transform infrared (FTIR) spectroscopy studies

**Figure 4:**
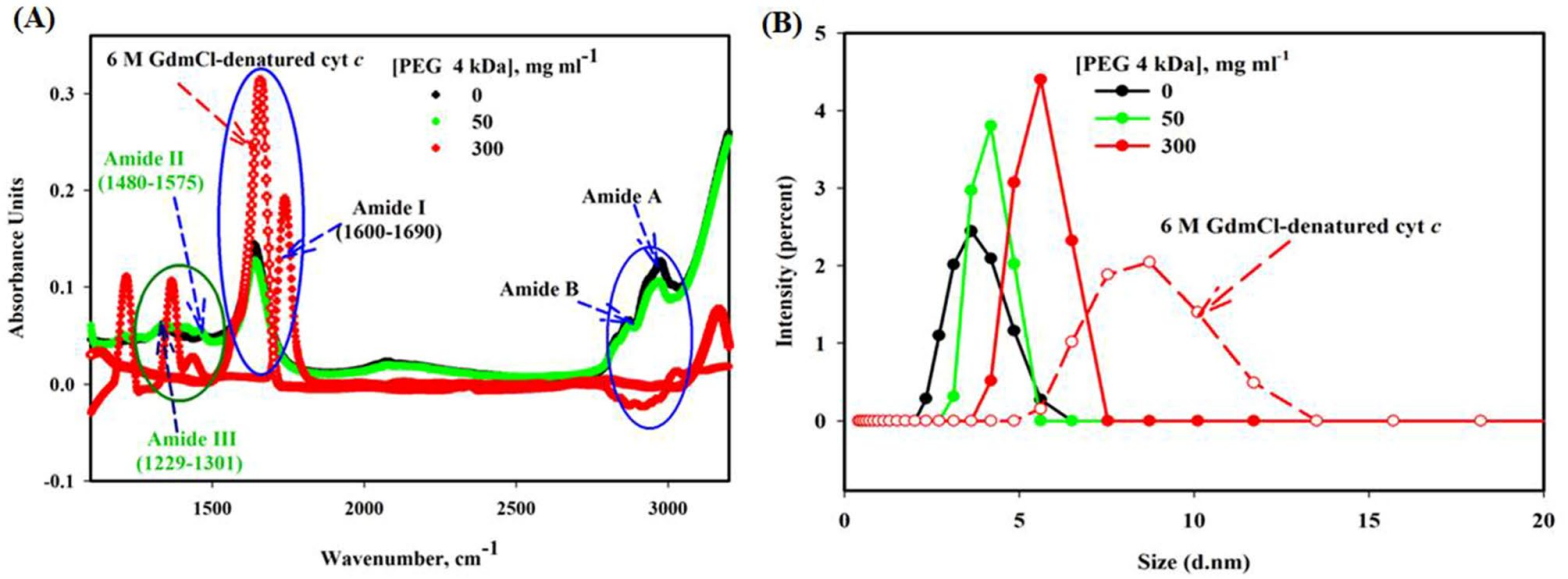
(**A**) FTIR spectra of cyt *c* in the presence of PEG 4 kDa at different concentrations (0, 50 and 300 mg ml^−1^) and GdmCl (6 M), shows various characteristic infrared bands of peptide linkage. (**B**) Particle size analysis with DLS confirms overall particle size (d.nm) of cyt *c* in the presence of PEG 4 kDa at different concentrations (0, 50, and 300 mg ml^−1^) and GdmCl (6 M).

Furthermore, the conformational changes as well as residue specific complex of cyt *c* without and with PEG 4 kDa and 6 M GdmCl were characterized by the system in Fourier transform infrared spectroscopy (FTIR). FTIR measurements of cyt *c* were carried out within milieu of various concentrations (0, 50 and 300 mg ml^−1^) of PEG 4 kDa and 6 M GdmCl (see Fig. 4A). The figure shows inconsequential change in absorbance when exposed to 50 mg ml^−1^ with small shift in wavenumber and drastic change in absorbance on exposure to 300 mg ml^−1^ of PEG 4 kDa with a large shift around wave number of 1200–1301 (amide region III) and 1480–1575 (amide region II) showing CN stretching and NH Bending, 1600–1730 cm^−1^ (amide I region) which shows C = O stretching and negative peak observed around the region 3000–3500 cm^−1^ shows NH-stretching^59^. Observations from the figure also show strong changes in absorbance and shifts around different wavenumber regions of the protein exposed to 6 M GdmCl.

### Dynamic light scattering studies

To measure the sizes in terms of hydrodynamic radius (*R*_h_) values of the native cyt *c* (in buffer), in the presence of PEG 4 kDa (50 and 300 mg ml^−1^) and exposed to 6 M GdmCl, dynamic light scattering (DLS) technique was exploited. The diameter values of each sample were measured by the software Zetasizer Ver. 7.13 of Malvern Panalyticalin nanometers (nm) and from that the hydrodynamic radius (*R*_h_) was calculated. The hydrodynamic volume (*V*_h_) values of each state is measured from its hydrodynamic radius (*R*_h_) using the relation, 4/3π (*R*_h_)^3,60^. Figure 4B shows that the hydrodynamic radius of cyt *c* increases from 1.65 nm (16.5 Å), in buffer to 1.84 nm (18.4 Å) exposed to 50 mg ml^−1^ of PEG 4 kDa, further, the size of *R*_h_ increases more than 50 percent i.e. 2.75 nm (27.5 Å) when exposed to 300 mg ml^−1^ of PEG 4 kDa. The hydrodynamic radius of cyt *c* in 6 M GdmCl, shown in this figure is equal to 3.85 nm (38.5 Å). The *R*_h_ and *V*_h_ values acquired for cyt *c* in different co-solvents (buffer, PEG 4 kDa and 6 M GdmCl) from DLS are given in the Table 2 in angstroms. Table S1 provides the significance of various characteristic infrared bands and hydrodynamic raddi (*R*_h_) and hydrodynamic volume (*V*_h_) of the protein to distinguish native protein from denatured and more compacted protein.

**Table 2:** *R*_h_ and *V*_h_ values of cyt *c* in the presence of different solvent conditions at pH 7.0 and 25 °C.

Protein solvent condition	(*R*_h_), (Å)	(*V*_h_), (Å)^3^
Buffer	16.5 (± 0.6)^#^	18,131 (± 176)^#^
50 mg ml^−1^ PEG 4 kDa	18.4 (± 0.86)^#^	26,080 (± 485)^#^
300 mg ml^−1^ PEG 4 kDa	27.5 (± 0.95)^#^	87,069 (± 720)^#^
6 M GdmCl	38.5 (± 4.5)^#^	238,918 (± 1420)^#^

^#^With each parameter signifies the mean error.

### Isothermal titration calorimetry studies

**Figure 5:**
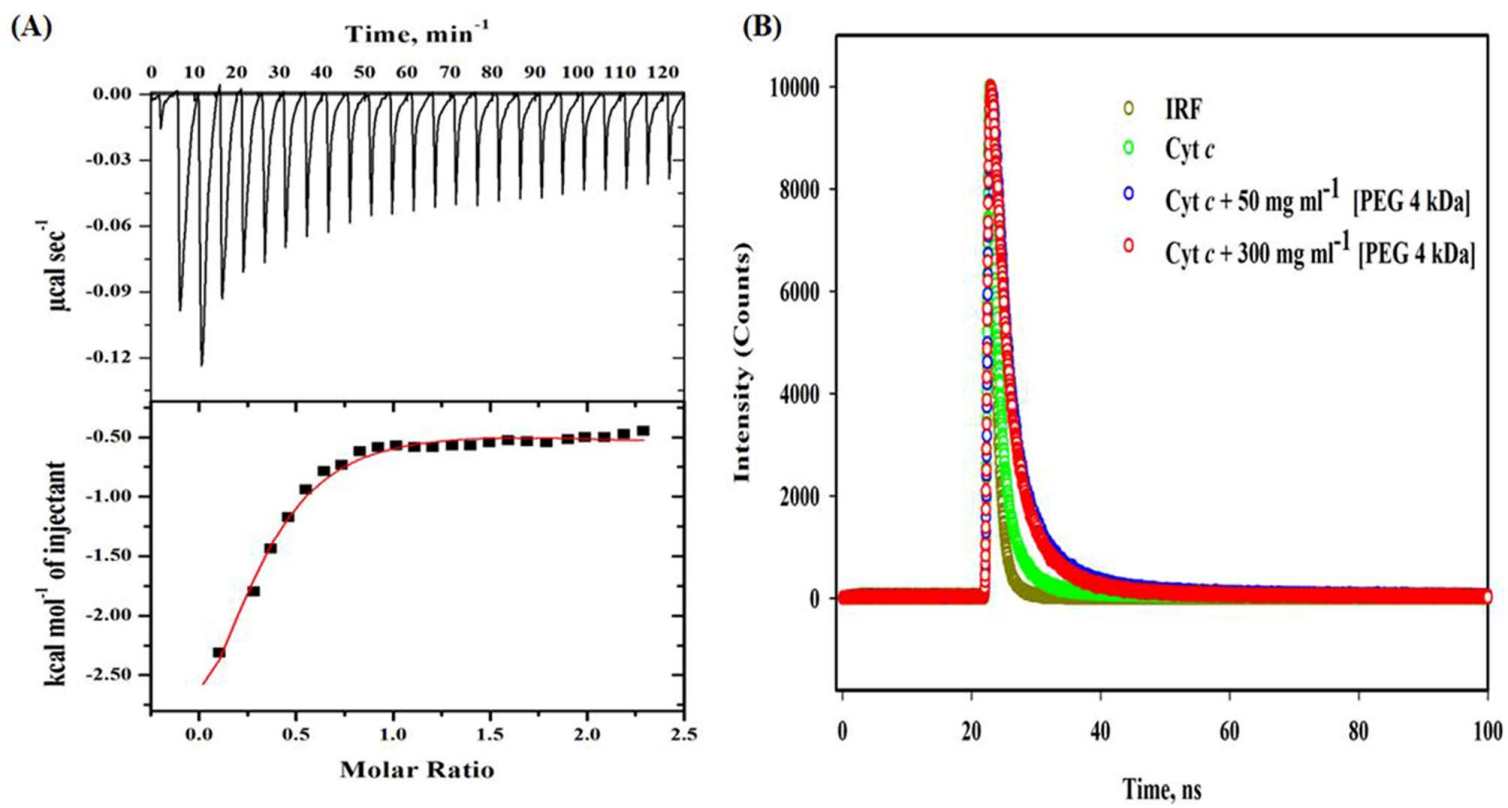
(**A**) Typical ITC thermogram of cyt *c* (20 μM) showing calorimetric response on successive injection of the PEG 4 kDa (600 μM) added to the reaction cell at pH 7.0 and 298 K (25 °C). (**B**) Time resolved fluorescence showing fluorescence decay of cyt *c* with increasing concentration of PEG 4 kDa along with lamp profile which shows an instrument response function (IRF) at pH 7.0 and 25 °C.

Structural distortion of cyt *c* in the milieu of the crowder has been proved, and to know the cause and chemical basis, the ITC measurements were made. In this experiment, PEG 4 kDa, which was loaded in the syringe, was titrated into the sample cell where cyt *c* was placed. The top section of the Fig. 5A give the raw data, power is plotted against time. Lower panel in this figure shows the raw data in power normalized to the injectant amount in kcal mol^−1^ against molar ratio of PEG 4 kDa injections. Table 3 provides the values of binding association constant (*K*_a_), equilibrium dissociation constant, *K*_d_, enthalpy (Δ*H*°) and entropy (Δ*S*°) change of PEG 4 kDa binding with cyt *c*. From the obtained primary parameters, secondary and considerable parameter i.e., free energy change (Δ*G*°) of the bi-molecular reaction was calculated using Eq. (2). Table 3 values signify that the Δ*G*° is highly negative and the bi-molecular reaction is spontaneous and the sum of changes of Δ*H*° is negative hence binding of PEG 4 kDa to the protein is exothermic.

**Table 3:** Binding parameters obtained from calorimetric measurements due to changes in cyt *c* on interaction with PEG 4 kDa at 298 K (25 °C) and pH 7.0.

Thermodynamic parameters (Units)	*K*_a_ (M^−1^)	*∆H*° (cal mol^−1^)	*∆S*° (cal mol^−1^ deg^−1^)	*∆G*° (cal mol^−1^)	*K*_d_ (M)
Step 1	48.7 × 10^3^ (± 4.8 × 10^3^)^#^	− 4075 (± 225)^#^	7.78	− 6.395 × 10^3^ (± 0.23 × 10^3^)^#^	0.021 × 10^–3^
Step 2	24.8 × 10^3^ (± 2.6 × 10^3^)^#^	7968 (± 720)^#^	46.8	− 1.219 × 10^3^ (± 0.72 × 10^3^)^#^	0.040 × 10^–3^
Step 3	46.0 × 10^3^ (± 5.03 × 10^3^)^#^	− 9539 (± 924)^#^	− 10.7	− 6.349 × 10^3^ (± 0.92 × 10^3^)^#^	0.022 × 10^–3^

^#^With each parameter signifies the mean error.

### Time resolved fluorescence

The shifts in strength and the changing of Trp emissions have the advantage of discovering the truth about the regional conformation changes in the Trp system and the essence of protein–ligand interactions^61^, time-resolved fluorescence spectroscopy, which is an analytical tool, may be used to analyze such changes in the protein^62^. Figure 5B shows the tryptophan fluorescence decay of cyt *c* (in buffer) and cyt *c* exposed to PEG 4 kDa (50 and 300 mg ml-1), at an excitation wavelength of 280 nm, provided with a precision of lamp profiling. The decay profiles were built from the triple exponential equation function (3)^63^. Table 4 provides reports of decay (including values of τ (ns) and α (%) and χ^2^) of the protein (in the buffer) and exposure of PEG 4 kDa.

**Table 4:** Parameters obtained from time resolved fluorescence spectra (excited at 280 nm) of pure cyt *c* and cyt *c*–PEG 4 kDa with different concentrations pH 7.0 and 25 °C^a^.

Sample	τ_1_ (ns)	τ_2_ (ns)	τ_3_ (ns)	α_1_ (%)	α_2_ (%)	α_3_ (%)	χ^2^	τ_avg._ (ns)	τ_0/_τ
Cyt *c* pure	5.94 (± 0.27)	1.5 (± 0.02)	0	0.094	0.090	0	1.09	5.07 (± 0.25)	1 (± 0.08)
Cyt *c* + PEG 4 kDa (50 mg ml^−1^)	2.6 (± 0.04)	9.2 (± 0.16)	0	0.87	0.133	0	1.07	7.32 (± 0.35)	0.69 (± 0.06)
Cyt *c* + PEG 4 kDa (300 mg ml^−1^)	3.49 (± 0.13)	16.6 (± 0.6)	0.51 (± 0.001)	0.19	0.02	0.79	1.06	5.74 (± 0.27)	0.88 (± 0.07)

^a^A ± with each parameter signifies the mean error.

### Molecular docking studies

**Figure 6:**
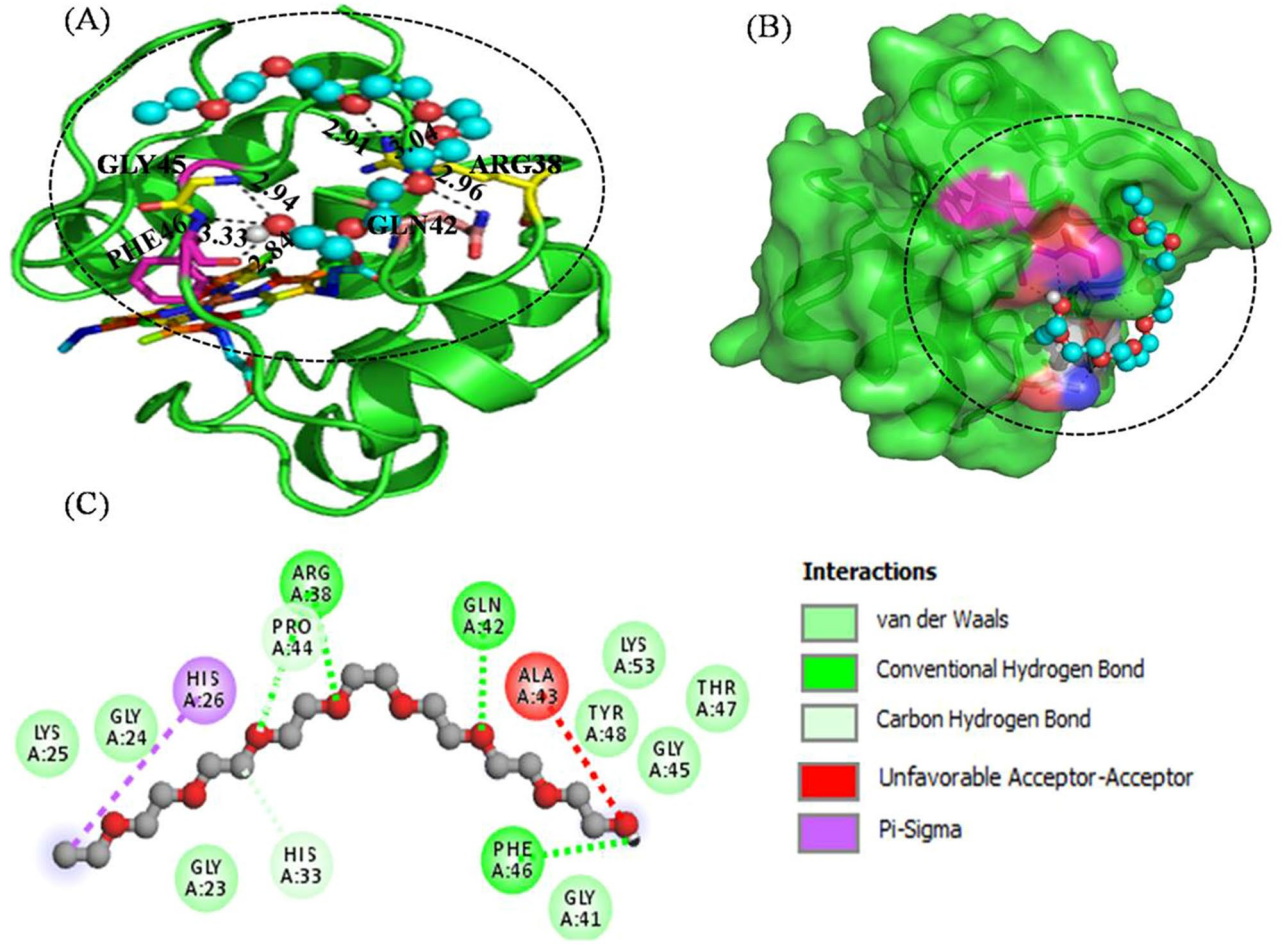
(**A**) Interactions of PEG 4 kDa (ball and stick model) with cyt *c* (cartoon model, green), (**B**) Surface view model and (**C**) 2D representation of various types of interactions of PEG 4 kDa with the amino acid residues of the protein.

The interactions studies were exploited to investigate the structural change or retention of heme group in cyt *c* when treated with PEG 4 kDa. To know the binding site residue of cyt *c* that interact with PEG 4 kDa, in silico studies and analysis of cyt *c* (PDB ID:1hrc) with the ligand molecule (PEG 4 kDa) was executed by molecular docking. PEG interacts through hydrogen bonding with bond distances of 2.84 Å with PHE46 (single h-bond), 2.96 Å with GLN42 (single h-bond), 3.19 and 2.94 Å with GLY45 (two h-bond) and 3.04 and 2.91 Å with ARG38 (see Fig. 6A). Figure 6B depicts the surface view of the protein with pocket binding site of PEG 4 kDa. Figure 6C gives the 2D representation of the amino acid residues showing different types of interactions (conventional H-bonding, vander Walls forces, carbon hydrogen bond, pi-sigma bond and unfavorable accepter-acceptor) with PEG 4 kDa. The in silico analysis reveals that feasible interaction occurs between PEG 4 kDa and cyt *c* with − 3.9 kcal mol^−1^ of binding energy.

## Discussion

The concentration dependent change due to PEG 4 kDa in the structure of cyt *c* using broad absorption spectra is shown in Fig. 1A,B. The figures shows that cyt *c* has several absorption bands around 280 nm (arises because of the aromatic side chains of Tyr and Trp residues), Soret region at 409–410 nm (due to heme) and an oxy-deoxy small band around wavelength range of 500–600 nm^47^. In pure cyt *c* (in buffer), sharp Soret band at 409 nm indicates the native condition of the protein^64^. This sharp peak appears because of the π–π* electron transition occurred in the heme, which is placed inside the hydrophobic pocket of the protein structured by its backbone during proper folding. The position and shape of Soret band exclusively is dependent on the iron atom’s geometric position in the heme^40,65^. The slight increase in intensity of Soret band at low concentration of PEG 4 kDa (50 mg ml^−1^) without red shift, reflects a small change in the chemical milieu of cyt *c* and on adding more concentrations of PEG 4 kDa (100, 150, 200 and 250 mg ml^−1^) such changes in the protein decreases and at highest concentration (300 mg ml^−1^) it overlaps to the native protein spectrum. There was no change observed in oxy-deoxy region band 500–600 nm, hence the protein was in oxidized form at all concentrations. However, Trp and Tyr environment changes from non-polar to polar environment (observed by *ε*_280_, absorption coefficient at 280 nm), which suggests that tertiary structure was perturbed at highest concentration (300 mg ml^−1^) without affecting heme-protein interaction. In contrast, at lower concentration of PEG 4 kDa (50 mg ml^−1^), it was observed heme gets displaced and causes increase in the absorbance without affecting the Trp and Tyr environment (see Fig. 1A,B). Previously it has been reported that PEG 4 kDa of small concentrations has little change without any shift in Soret absorption and oxy-deoxy band in cyt *c*^31^. The studies claim that PEG 4 kDa promotes auto-oxidation of cyt *c*, however changes are observed to be insignificant^31^. In addition, the study proposed a model showing that heme is dislocated due to dehydration of some surface exposed hydrophobic residues i.e., ILE81 and VAL83; however spectroscopic techniques showed small changes^31^ and are comparable to our results at lower concentrations of the crowder. This confirms that conformational change showed by heme is insignificant and may be momentary at small concentrations of PEG 4 kDa and are retained as the crowded condition is increased to higher concentration.

To confirm the changes by absorption spectroscopy (Fig. 1A,B), effect of PEG 4 kDa was observed on the structure of cyt *c* by using another probe, i.e. near-UV CD measurements (for gross tertiary interactions) (see Fig. 2B), Soret-CD (Heme-protein, heme-PHE, and MET-Fe interactions) (see Fig. 2A) and the far-UV CD spectra were taken to measure the effect of PEG 4 kDa on the secondary structure of the protein (see Fig. 2C). The near-UV CD spectrum of cyt *c* (see Fig. 2B) showed two negative CD signals around wavelengths of 282 and 289 nm allocated to a rigid packing of structure (tertiary) in the environment of Tyr and Trp59 residue and because of Trp59 interaction with heme via one of its propionate group^66^. Figure 2B, which depicts near-UV CD spectra of the protein in the presence of various PEG 4 kDa concentrations (0, 50 and 300 mg ml^−1^) and from this data it was observed that 50 mg ml^−1^ has no effects on the Trp and Tyr environment, however, at 300 mg ml^−1^ PEG 4 kDa, [*θ*]_282_ and [*θ*]_289_ values shows maximum decrease in CD signals which indicates loss of the tertiary structure. The observations from near-UV CD supports near-UV absorption studies of cyt *c* exposed to crowder where change in Trp and Tyr environment was observed significant at higher concentration of the crowder. The near-UV CD also shows spectrum of denatured-cyt *c* which shows complete disappearance of the [*θ*]_282_ and [*θ*]_289_ values. The figures (Figs. 1B, 2B) shows that the tertiary structure of cyt *c* is destabilized maximum at 300 mg ml^−1^ of PEG 4 kDa, however there was no significant change when protein was treated by 50 mg ml^−1^ of PEG 4 kDa. Soret CD is a suitable probe to investigate the alterations in heme environment by measuring the changes at 405 nm, [*θ*]_405_; further, [*θ*]_416_ shows the interaction strengths of PHE82-heme and MET80-Fe^48,49^. Soret CD measurements revealed that there was significant increase in CD signal (positive peak) of cyt *c* spectra at [*θ*]_405_ when exposed to PEG 4 kDa of 50 mg ml^−1^, however change was insignificant due to high concentration of PEG 4 kDa i.e. 300 mg ml^−1^ (see Fig. 2A). And the change observed at [*θ*]_416_ shows a shift from (416 nm to 420 nm) in presence of 50 mg ml^−1^ PEG 4 kDa with a small increase in the ellipticity. It has been reported that any variation in these wavelengths (405 nm & 416 nm) suggests structural transitions in the protein^49^. It can be observed from Soret-absorption and Soret-CD (Figs. 1A,B, 2A), respectively) that the higher concentration of PEG 4 kDa has small affect on the heme-protein interactions than small concentrations which may be due to dehydration of surface exposed hydrophobic residues i.e., ILE81 and VAL83 as reported earlier also^31^. Recently published work from our group observed that PEG 400 Da (low molecular size PEG) at higher concentrations affects interaction between heme and protein in cyt *c* and Mb to greater extent due to soft interaction and results different intermediate states^4,10^. So, from this it can be confirmed that effects on the protein’s heme environment and its structure in similar conditions may be also protein, PEG and PEG concentration dependent.

As it is well known about the far-UV CD, which is an insightful probe to determine the secondary structural changes in the proteins^53^, so was explored to monitor the PEG 4 kDa effects in the presence of different concentrations (0, 50, 100, 150, 200, 250 & 300) and 6 M GdmCl on secondary structure of cyt *c* (Fig. 2C). The far-UV CD spectrum of the protein in buffer (Fig. 2C) has comparable signatures at 222 and 208 nm that had been reported earlier, which are characteristics of an all α-protein^54,67,68^. From the figure it is now confirmed that the native CD spectrum is perturbed with increasing concentration of PEG 4 kDa and it was observed that about 50% of the helical content is lost due to higher concentration of PEG 4 kDa. CD technique is not only used to observe the structural changes in proteins but has been used for interaction studies also in the presence of different ligands, which shows structural loss^69–71^. So from the CD measurements one can suggest that there is an interaction between cyt *c* and PEG 4 kDa that affect the structural characteristic of the protein. Table 1 gives percentage values of secondary structure content (α-helical) of the protein in buffer, in PEG 4 kDa and 6 M GdmCl, which were estimated from the values at [*θ*]_208_ and [*θ*]_222_, using equations of Greenfield and Fasman^38^ and Morrisett et al.^39^, respectively. The values given in the table showed that helical content of cyt *c* is approximately 50% retained of in PEG 4 kDa of 300 mg ml^−1^.

The intrinsic fluorescence of aromatic residues in proteins has long been exploited to monitor changes induced by temperature, pH, chemical denaturants and pressure for unfolding/refolding of proteins^10,72–74^. Besides unfolding/folding, the technique has also been used for interaction studies^75,76^. The characteristics of Trp residues in fluorescence particularly are deftly insightful probe to monitor the protein structure changes, while the low quantum yields of other aromatic amino acids (Phe and Tyr) are fairly less valuable for such analysis^77^. The tryptophan residue (Trp59), which is ~ 10 Å away from the heme in the wild type cyt *c*, is largely quenched due to its fluorescence resonance energy transfer of the heme close to it^73^. The environment of Trp59 and Tyr groups changes to polar from non-polar, when cyt *c* is exposed to PEG 4 kDa which results in decreasing in quenching and increase in the intensity as the PEG 4 kDa concentration is raised up from 0 to 300 mg ml^−1^ (see Fig. 3A) and hence perturbs the structure. The inset of Fig. 3A showed tertiary structure is not perturbed completely, which supports near-UV CD (Fig. 2B). These observations suggests that the heme and Trp environment changes, hence the structure of the protein can be said to be perturbed by PEG 4 kDa at higher concentrations, similar to that of reported studies^10,31,78–80^. Table S1 provides the significance of various probes, which monitors the change in the environment of aromatic amino acids, heme, various heme-protein residue interactions and secondary structural changes.

To detect the hydrophobic patches exposed in PEG 4 kDa-induced denatured-cyt *c*, ANS fluorescence studies were carried out. In polar solvents like buffers, ANS has insignificant emission spectrum. When the dye is transferred to non-polar solvents, the emission spectrum enhances significantly with hypsochromic shift (to shorter wavelength)^58^. The fluorescent intensity of the protein (cyt *c*) treated by PEG 4 kDa enhanced with a lower wavelength shift (Fig. 3B); λ_max_ of fluorescence of ANS shifted from higher wavelength of 516 nm (the protein in buffer) to lower wavelength of 507 nm (the protein in 50 mg ml^−1^ PEG 4 kDa) and to lowest wavelength of 500 nm (the protein in 300 mg ml^−1^ PEG 4 kDa). These remarks led us to infer that the hydrophobic patches are less exposed to the polar environment at lower concentration while they are exposed dominantly due to higher concentration of PEG 4 kDa and binds with ANS, as an evident increase in the fluorescence intensity occurs with greater shift towards lower wavelengths. The protein in buffer and in 6 M GdmCl do not binds to ANS (Fig. 3B), because in foremost case the hydrophobic patches are hidden and in later case hydrophobic patches are lost^14^.

From the spectroscopic techniques (absorption, CD and fluorescence), it is confirmed that the cyt *c* structure get perturbed when treated with PEG 4 kDa at pH 7.0 and 25 °C and the protein has hydrophobic patches on the surface in the presence of PEG 4 kDa which confirms protein is in intermediate state (half denatured). To know the mechanism and means of this perturbation, additional comprehensible techniques were used which confirmed the interaction between the protein and the crowder molecule.

Fourier transform Infrared (FTIR) is one of the excellent experimental spectroscopic techniques exploited formerly for the investigating and monitor the changes in secondary structure content of polypeptides and proteins^81,82^. There are some reports where the FTIR has been applied to observe crowding effect over proteins^5,83–85^, and for the interaction studies of proteins with ligands^40,86,87^. Table S1 provides the significance of various characteristics infrared bands of the protein (peptide linkages) used to monitor the changes in the protein exposed to different environment (solvent conditions, pH and temperature change etc.). Figure 4A showed the infrared spectra of cyt *c* recorded against the wave number function, treated with the various concentrations of PEG 4 kDa in mg ml^−1^ (0, 50 and 300). The result of this figure has shown that peak positions of the amide bands and the intensity are affected at higher concentration of PEG 4 kDa and 6 M GdmCl. The most intense absorption band is shown around 1650–1690 cm^−1^ (due amide I vibrations) by the cyt *c* in buffer, however the absorbance of the protein is affected when treated with 300 mg ml^−1^, with large shift in wavenumber and small changes were observed because of 50 mg ml^−1^ of PEG 4 kDa. These vibrations bring generally the amide C=O group stretching^82^. The amide II band, region around 1480–1575 cm^−1^ and the amide III region around 1229–1301 cm^−1^ are mostly because of the alteration in the plane of N−H bending vibration together of peptide bond C−N stretching vibrations. The FTIR data also showed that the amide II band around 1570 cm^−1^ lessens considerably, whereas the absorbance intensity of the amide II′ band around 1444 cm^−1^ enhances and the amide III region also showed that absorbance is affected due to 300 mg ml^−1^ of PEG 4 kDa. Moreover, the region around 3000–3500 cm^−1^ includes amide A and amide B region which is due to NH stretching^59^, showed comprehensible negative peaks which decreased absorbance within region on increasing the [PEG 4 kDa]. The FTIR observations suggested that the protein structure was affected when exposed to PEG 4 kDa at higher concentrations and this change may be due to interaction of PEG 4 kDa.

Moreover, size distribution measurements of cyt *c* exposed to a choice of solvent conditions (buffer, [PEG 4 kDa] and 6 M GdmCl) were observed (see Fig. 4B). DLS is a very significant means to study about the diffusion behavior of macromolecules in solvent medium. The diffusion coefficient and the size, which is analyzed from the technique, depend on the shape and size of macromolecule^88^. Diameter of each sample in the presence of PEG 4 kDa was analyzed using software Zetasizer Ver. 7.13 of Malvern Panalytical and plotted as Intensity versus Diameter (nm). The obtained results in diameter were converted into hydrodynamic radii (*R*_h_). It was observed that *R*_h_ value of wild type cyt *c* (in the buffer) was 1.65 nm (16.5 Å) (see Table 2), which is nearly equal to the value reported^10,57,89^. Table 2 reports the *R*_h_ and *V*_h_ of the protein in each solvent condition. The *R*_h_ of cyt *c* increases from 1.65 nm (16.5 Å) to 1.84 nm (18.4 Å) when exposed to 50 mg ml^−1^ of PEG 4 kDa; further, more than 50 percent increase in the size occurred i.e., 2.75 nm (27.5 Å) due to 300 mg ml^−1^ of PEG 4 kDa. The calculated *R*_h_ value of the protein exposed to 6 M GdmCl was equal to 3.85 nm (38.5 Å). The *V*_h_ of cyt *c* treated with 50 mg ml^−1^ of PEG 4 kDa was observed to be about 1.5 times greater than of native state protein and the protein treated with 300 mg ml^−1^ of PEG 4 kDa was observed to be about 3.5 times greater than native protein. The volume enlarged of the protein exposed to PEG 4 kDa confirms that the protein gets denatured and PEG interacts with the protein. Table S1 provides the significance of DLS and parameters (*R*_h_ and *V*_h_) estimated from the technique, which monitors the change in the size of protein in various solvent conditions and its existence of interaction with co-solutes.

Moreover, ITC, time resolved fluorescence and computational studies were carried out to confirm the above observations. ITC analyzed data, which showed that the crowder binds with the protein, for which different models were used, however the raw data outcomes were best fitted by 3-step sequential binding site model to get final results (see Fig. 5A). The value of stoichiometry “n” can be obtained directly from the technique for one-site (1:1) and two-site binding model (1:2), however in sequential binding where ligands bind may have independent (more than 2 molecules bind at different sites) or identical sites (more than 2 molecules bind at the same site) on the protein, it is intricate to know the actual sites where ligand molecules bind with protein^90^. From the figure determination of the stoichiometry “*n*” of the bi-molecular interaction can be observed directly, which is the equivalence point of the molar ratio and from Fig. 5A (lower panel) it can be guesstimated that the stoichiometry is greater than 0.3 (which is equivalence point of the molar ratio). To recall few studies of ITC thermograms of few proteins with ligands, which were best described by sequential binding site models^91–94^. Calorimetric measurement is just a method which presents the thermodynamic parameters directly related with an interaction, as the enthalpy change presents the probe, which defines the degree of an interaction^95^. The raw data output of the interaction between cyt *c* and PEG 4 kDa and change in enthalpy values were enumerated in Table 3. These values confirm bi-molecular reaction, which is exothermic in nature at pH 7.0 and 298 K (25 °C). The values of change in free energy (Δ*G*°) are negative which means the intermolecular interaction (cyt *c*-PEG 4 kDa) is spontaneous shows good binding. ITC presents a best route that shows comprehensive description of bimolecular equilibrium interactions via thermodynamically. The calorimetric change in enthalpy and the equilibrium binding constant, *K*_a_ (equal to the inverse of the dissociation constant, *K*_d_, for bi-molecular reaction) can be known through this calorimetric measurements of bimolecular interaction where one molecule is titrated into the other^95^. The *K*_d,_ values evaluate rank of the strength between two molecules in interactions.

Binding affinity (*K*_a_) is a key to understand the appreciation of the intermolecular interactions which drives the biological processes, structural biology and structure–function relationships^95^. Binding affinity is affected by inter-molecular weak contacts usually non-covalent interactions, for example: electrostatic interactions, van der Waals forces, hydrogen bonding and hydrophobic interaction.

From the time resolved fluorescence experiment as shown in Fig. 5B and Table 4, it should be noted that with the increase in [PEG 4 kDa], the average lifetime of cyt *c* increases from 5.07 ns (in buffer) to 7.32 ns (in 50 mg ml^−1^ PEG 4 kDa) and at 300 mg ml^−1^ PEG 4 kDa, it decreases to 5.74 which is also larger value in comparison to that of the cyt *c* in buffer. This increase essentially signifies the metal enhanced fluorescence is active at the high [PEG 4 kDa]^40^. The electron transfer achieved from Trp excitation and exposure of Trp to exterior milieu indicate the interaction of PEG 4 kDa with protein which enhances fluorescence and causes changes in lifetime and lead to the local conformational change in the protein^96^. The different *τ* (*τ*_0_, *τ*_1_, *τ*_2_, *τ*_3_, average lifetime (*τ*_avg._), and α values (α_1_, α_2_ and α_3_) of each sample are given in Table 4. The *τ*_0_/*τ* value is equal to 1 within errors for the native protein, which is quenched due to heme. It is observed from Fig. 3A that fluorescence increases and quenching decreases as the concentration of PEG 4 kDa is increased. However, *τ*_0_/*τ* value for cyt *c* when exposed to the crowder is less than 1 and *τ*_0_/*τ* versus [PEG 4 kDa] can’t be flat because the PEG 4 kDa increased fluorescence intensity (see Fig. 3A) does not showed quenching, so the structure of the protein exposed to the crowder get perturbed due to interaction^41^. From the spectroscopic and interaction studies one can say that the protein (cyt *c*) destabilization is caused because of interaction of PEG 4 kDa without affecting the heme environment.

The techniques including ITC, FTIR, circular dichroism and time resolved fluorescence confirms that PEG 4 kDa interacts with cyt *c*. In addition from the absorption studies (Fig. 1) and Soret-CD (Fig. 2A) showed heme retention takes place even at higher concentration of PEG 4. To know the pocket site on the protein where PEG 4 kDa binds, the computational studies (molecular docking) was executed. Figure 6 displays the various weak interactions of the ligand with receptor shown in dashes-and bond-distances are labeled. The interaction of ligand with the residues of the protein includes phenylalanine 46 (single bond of 2.84 Å), glycine 45 (two bonds of 3.33 and 2.84 Å), arginine 38 (two bonds of 3.96 Å and 2.91 Å) and glutamine 42 (single bond of 2.96 Å). It was the best pose of 9 binding sites, which shows feasible interaction between PEG 4 kDa and cyt *c* of binding energy of -3.9 kcal mol^−1^. Figure 6B showed the surface view of the protein that exhibits binding pocket site for PEG 4 kDa on its surface. Figure 6C showed the two-dimensional representation of various types of interactions between PEG 4 kDa (in ball and socket) and various residues of cyt *c*, PEG 400 Da (smaller than PEG 4 kDa) which interacts with heme and GLN42, TYR48 and LEU35 of the protein through hydrogen bonding which perturbed the structure by changing heme-TRP59, MET80-Fe and PHE82-heme distance, results in heme disruption without affecting the secondary structure and yields molten globule state^10^. The both PEGs (PEG 400 Da and PEG 4 kDa) bind with different residues except GLN42, which confirms it may be the active site for PEGs to bind in cyt *c*. The residues of cyt *c* ARG38, PHE46, GLU42, GLY45 binds with PEG 4 kDa and TYR48 and LEU35 bind with PEG 400 Da and GLN42 in both confirms the binding site is similar and due to size difference in the PEGs, residues are interacting greater to PEG 4 kDa. In addition, earlier reports had proposed a model where they showed that heme was dislocated due to dehydration of some surface exposed hydrophobic residues i.e., ILE81 and VAL83 in the presence of PEG 4 kDa; however their spectroscopic techniques showed no significant changes at such concentrations^31^ and were observed comparable to our outcomes at lower concentrations of the crowder.

**Figure 7:**
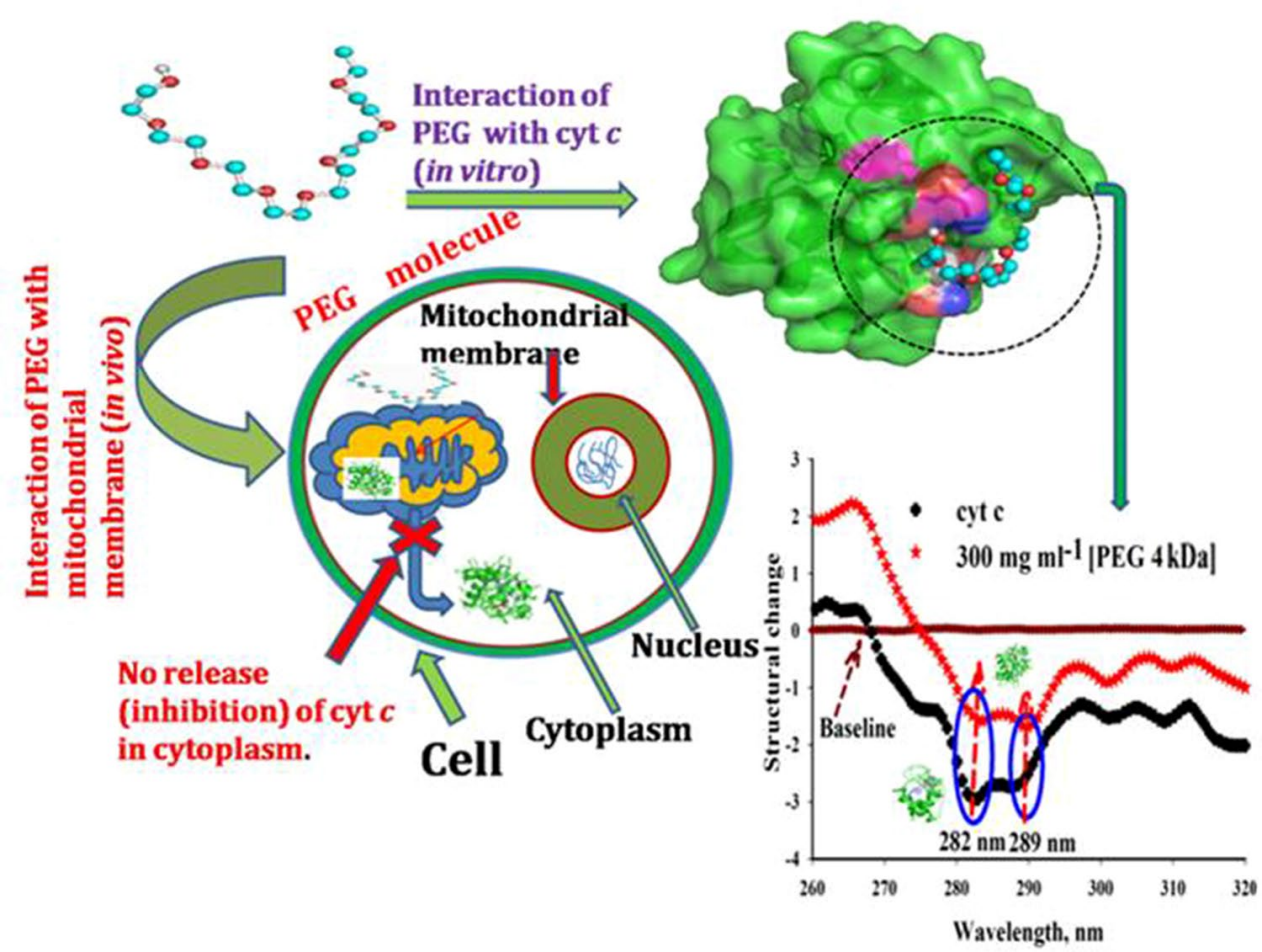
The visual representation of comparative effect of PEG 4 kDa on cyt *c *in vitro and in vivo conditions due to interaction.

It has been reported that cyt *c* plays important role in transfer of electrons from cyt bc_1_ complex to cyt *c* oxidase in respiratory chain in mitochondria and also plays a key role in apoptosis, where it is released to cytosol and triggers caspase cascades^97,98^. The prosthetic group (heme) is covalently bound by two cysteine residues of the protein through their sulphur atoms and coordinated by HIS18 and MET80 in the native form^99^. The interesting fact is that MET80-heme dissociation induces peroxidase activity and leads to the oxidation of cardiolipins, in turn, leading to release of pro-apoptotic factors^52^. Our studies which showed (Figs. 1B, 2A) that heme-MET80 interactions do not get perturbed (stabilizes the interaction of the C-terminal helical region with the N-terminal region^100^), however secondary and tertiary structure (not completely) are perturbed in the presence of PEG 4 kDa, confirms the earlier studies which showed that PEG 2 kDa on interaction with mitochondrial membrane inhibit the release of cyt *c* (pro-apoptotic factors) in the cytoplasm, lacks peroxidase activity^8,20^ (portrayed visually in Fig. 7).

Conversely, in this study, the spectroscopic studies showed that the heme is intact at its place with the protein in the presence of PEG 4 kDa even when the both the secondary and tertiary structure is lost but not completely. Intactness of the heme group is also confirmed by the computational studies (see Fig. 6), which shows that heme group is not involved in the interaction with PEG 4 kDa. The in vitro spectroscopic approaches confirmed that conformational changes occurred in heme are insignificant due to small concentrations of PEG 4 kDa, however are retained as the crowded condition is increased to higher concentrations. Figure 6 showed that PEG 4 kDa interacted with various amino acid residues on the protein which may be the cause of change in heme-globular interactions at low concentrations. From the results it is confirmed that the change in the heme-globular and overall structure of the protein is crowder as well as concentration dependent.

PEG and (NH_4_)_2_HPO_4_ had been observed to change or shift the trimeric linear form of cyt *c* towards cyclic structure, monitored by small-angle X-ray scattering^100^. The receptor and ligand are treated as rigid bodies, but dislocation of a few number of residues make space for the ligand to bind, which are enough to be taken into consideration, for the changes in the protein^101^. From the spectroscopic studies and interaction studies it can be said that the structural changes in cyt *c* occurs due to PEG 4 kDa binding (portrayed visually in Fig. 7) and the changes in the protein are crowder-concentration dependent.

## Conclusion

The spectroscopic techniques showed that the secondary and tertiary structure of cyt *c* was observed to be incompletely lost in the presence of PEG 4 kDa at higher concentrations with insignificant change in heme and unaffected heme-residue (MET80 and PHE82) interactions at high concentrations. This confirms that change in the heme-globular and overall structure of the protein is crowder’s size as well as concentration dependent. The interaction studies showed that the structural perturbation in the protein occurred due to binding of the crowder (PEG 4 kDa). From the previously reported studies and our results reassure that PEGs are now no more inert molecules and they interact with various proteins by “soft and specific” interactions. The site of binding with good binding affinity in cyt *c* for PEG 400 Da and PEG 4 kDa is similar and GLN42 residue interacts with both. Met80-Heme interactions were not found perturbed at each concentrations, which confirms that PEG 4 kDa may be acted as the inhibitor of pro-apoptotic cell death factor (cyt *c*) similar to PEG 2 kDa which has been reported to interact with mitochondrial membrane and boosts mitochondrial function which in turn inhibits cyt *c* for releasing into cytoplasm^8,20^. From the spectroscopic and interaction investigations of in vitro, the authors are motivated to design studies of in vivo by treating cancer cell lines using various concentrations of PEG 4 kDa.

## Supplementary Information

Supplementary Information
