# Supplementary material for: Interaction of polyethylene glycol with cytochrome c investigated via in vitro and in silico approaches
Source: Sci Rep. 2021 Mar 19;11:6475. doi: 10.1038/s41598-021-85792-4 (PMC7979836; doi:10.1038/s41598-021-85792-4)
Supplement: Supplementary file 1 — Supplementary Information [file 41598_2021_85792_MOESM1_ESM.docx]

**Interaction of polyethylene glycol with cytochrome c investigated via *in vitro* and *in silico* approaches**

Zahoor Ahmad Parray^1^, Faizan Ahmad^1^, Mohamed F. Alajmi^2^, Afzal Hussain^2^, Md. Imtaiyaz Hassan^1^, and Asimul Islam^1*^

^1^Centre for Interdisciplinary Research in Basic Sciences, Jamia Millia Islamia, Jamia Nagar, New Delhi 110025, India.

^2^Department of Pharmacognosy College of Pharmacy, King Saud University, Riyadh, Saudi Arabia.

**^*^Corresponding Author:**

**Dr. Asimul Islam**

**Member, National Academy of Sciences, (NASI)**

Centre for Interdisciplinary Research in Basic Sciences

Jamia Millia Islamia

Email: [aislam@jmi.ac.in](mailto:aislam@jmi.ac.in)

Mobile No: 00919312812007

**Table S1: Various probes from different techniques used to monitor the structural changes of cyt *c*.**

| **Techniques** | **Probes** | | | **Importance of probes** | | | | | **Ref.** | |
| --- | --- | --- | --- | --- | --- | --- | --- | --- | --- | --- |
| UV-visible absorption spectroscopy | *ε*_280_ | | | Absorption arises due to the aromatic side chain of Tyr and Trp residues; and change in the environment leads changes in absorbance within this range (275-290 nm), which is an indicator of change in tertiary structure. | | | | | [^1-4^](#_ENREF_1) | |
|  | *ε*_409_ | | | Monitor the metallo**-**proteins (with prosthetic groups), monitors change in the heme-globular interaction, change in iron spin state and helps researchers to investigate and observe the tertiary structural modifications. | | | | |  |  |
|  | *ε*_500–600_ | | | This is an oxy-deoxy band (small) around 500–600 nm region, confirms protein whether it is in reduced form (two bands) and/or in oxidized form (single band). | | | | |  |  |
| Circular Dichroism (CD) spectroscopy | [*θ*]_222_ and [*θ*]_208_ | | | They are two strong CD signals in cyt *c* which define the characteristics of all α-protein. These help in monitoring helical content of the proteins, protein-ligand interaction and distinguish mutants from wild proteins. | | | | | [^5-9^](#_ENREF_5) | |
|  | [*θ*]_282_ and [*θ*]_289_ | | | are two strong CD signals in cyt *c* in near-UV region monitors change in the environment of aromatic amino acids, which results change in tertiary structures in the presence of additive. | | | | |  |  |
|  | [*θ*]_405_ | | | It is an excellent probe to know changes in heme environment and its interaction with the protein via various amino acids. | | | | | [^10^](#_ENREF_10)^,^[^11^](#_ENREF_11) | |
|  | [*θ*]_416_ | | | It is an excellent probe for observing the interaction strength of Met80-Fe and Phe82-heme bonds. | | | | | [^12^](#_ENREF_12)^,^[^13^](#_ENREF_13) | |
| Fluorescence spectroscopy | Intrinsic | | *F*_342_ | | The characteristics of Trp residues in fluorescence particularly are deftly insightful probe to monitor the protein structure changes. Trp59 which is ~10 Å away from the heme in the wild type cyt c, is largely quenched due to its fluorescence resonance energy transfer of the heme close to it and change in the fluorescence intensity and/or shift leads decrease in quenching, hence perturbation of tertiary structure. | | | [^14^](#_ENREF_14)^,^[^15^](#_ENREF_15) | |  |
|  | ANS binding | | Increase in fluorescence intensity (*F*_516_) with wavelength (*λ*) shift. | | | The surface exposed hydrophobic patches on the protein can be probed using ANS binding, showing increase in the fluorescence intensity with a blue shift in *λ*, helps in the characterization of intermediate states (molten globule and pre-molten globules) and distinguishes mutant from wild protein. | | | [^8^](#_ENREF_8)^,^[^16^](#_ENREF_16) | |
| Fourier transform infrared (FTIR) spectroscopy | | Oldest experimental technique exploited for analysis of secondary structure of polypeptides and proteins. The technique is exploited for the interaction studies of proteins with ligands and also applied to observe effect of crowding over proteins. The change in absorbance and shift in wavenumber in the bands given monitors the changes in the protein. | | | | | | | [^17-23^](#_ENREF_17) | |
|  |  | amide region III  (1200-1301) and 1480-1575 (amide region II) | | | | | Occurs mainly due to the change in plane of N−H bending vibration coupled with the C=N stretching vibration of the peptide bond | |  |  |
|  |  | amide I (1650-1690 cm^−1^) | | | | | The most intense absorption band by proteins, shift and change in wave number around signifies change in the secondary and this vibration involves mostly due to the stretching of the amide C=O group | |  |  |
|  |  | amide A and amide B region (around 3000-3500 cm^−1^) | | | | | This band is due to NH stretching. | |  |  |
| Dynamic light scattering (DLS) | | Hydrodynmaic radii *(R*_h_) and hydrodynamic volume (*V*_h_). | | | | | The technique is exploited in biological laboratories to detect aggregates in the solutions of macromolecules for studying diseases caused due to protein aggregation. Besides exploited for size determination and monitoring ligand-protein interactions.  *R*_h_ for cyt *c* = ~16 Ǻ, on compaction the size decreases and on denaturation the size of the protein increases in the presence of additive. | | [^24^](#_ENREF_24)^,^[^25^](#_ENREF_25) | |

**References**

1 Dixon, M., Hill, R., Keilin, D. The Absorption Spectrum of the Component c of Cytochrome. *Proceedings of the Royal Society of London. Series B, Containing Papers of a Biological Character* **109**, 29 (1931).

2 Schmid, F.-X. in *Biological Macromolecules: UV‐visible Spectrophotometry* (ed Wiley Online Library) (2001).

3 Myer, Y. P. Conformation of cytochromes. III. Effect of urea, temperature, extrinsic ligands, and pH variation on the conformation of horse heart ferricytochrome c. *Biochemistry* **7**, 765-776, doi:10.1021/bi00842a035 (1968).

4 Drew, H. R. & Dickerson, R. E. The unfolding of the cytochromes c in methanol and acid. *J Biol Chem* **253**, 8420-8427 (1978).

5 Morrisett, J. D., David, J. S., Pownall, H. J. & Gotto, A. M., Jr. Interaction of an apolipoprotein (apoLP-alanine) with phosphatidylcholine. *Biochemistry* **12**, 1290-1299 (1973).

6 Moza, B. *et al.* A unique molten globule state occurs during unfolding of cytochrome c by LiClO4 near physiological pH and temperature: structural and thermodynamic characterization. *Biochemistry* **45**, 4695-4702, doi:10.1021/bi052357r (2006).

7 Moosavi-Movahedi, A. A. *et al.* Electrochemical evidence for the molten globule states of cytochrome c induced by N-alkyl sulfates at low concentrations. *Journal of Protein Chemistry* **22**, 23-30, doi:Doi 10.1023/A:1023011609931 (2003).

8 Khan, S. H. *et al.* Effect of conservative mutations (L94V and L94I) on the structure and stability of horse cytochrome c. *Arch Biochem Biophys* **633**, 40-49, doi:S0003-9861(17)30458-7 [pii]10.1016/j.abb.2017.08.015 (2017).

9 Greenfield, N. & Fasman, G. D. Computed circular dichroism spectra for the evaluation of protein conformation. *Biochemistry* **8**, 4108-4116 (1969).

10 Hamada, D. *et al.* Role of heme axial ligands in the conformational stability of the native and molten globule states of horse cytochrome c. *J Mol Biol* **256**, 172-186, doi:S0022-2836(96)90075-3 [pii]10.1006/jmbi.1996.0075 (1996).

11 Myer, Y. P. Ferricytochrome c. Refolding and the methionine 80-sulfur-iron linkage. *J Biol Chem* **259**, 6127-6133 (1984).

12 J. Pielak, G., Oikawa, K., Mauk, G., Smith, M. & M. Kay, C. *ChemInform Abstract: Elimination of the Negative Soret Cotton Effect of Cytochrome c by Replacement of the Invariant Phenylalanine Using Site-Directed Mutagenesis*. Vol. 17 (1986).

13 Santucci, R. & Ascoli, F. The Soret circular dichroism spectrum as a probe for the heme Fe(III)-Met(80) axial bond in horse cytochrome c. *J Inorg Biochem* **68**, 211-214, doi:S0162-0134(97)00100-1 [pii] (1997).

14 Chen, R. F. Fluorescence Quantum Yields of Tryptophan and Tyrosine. *Analytical Letters* **1**, 35-42, doi:10.1080/00032716708051097 (1967).

15 Das, T. K., Mazumdar, S. & Mitra, S. Characterization of a partially unfolded structure of cytochrome c induced by sodium dodecyl sulphate and the kinetics of its refolding. *Eur J Biochem* **254**, 662-670 (1998).

16 Stryer, L. The interaction of a naphthalene dye with apomyoglobin and apohemoglobin. A fluorescent probe of non-polar binding sites. *J Mol Biol* **13**, 482-495, doi:S0022-2836(65)80111-5 [pii] (1965).

17 Pinheiro, T. J. The interaction of horse heart cytochrome c with phospholipid bilayers. Structural and dynamic effects. *Biochimie* **76**, 489-500, doi:0300-9084(94)90173-2 [pii] (1994).

18 Smith, B. C. Fundamentals of Fourier Transform Infrared Spectroscopy. *2nd Edition: Boca Raton:CRC Press*, 207, doi:https://doi.org/10.1201/b10777 (2011).

19 Krimm, S. & Bandekar, J. Vibrational spectroscopy and conformation of peptides, polypeptides, and proteins. *Adv Protein Chem* **38**, 181-364 (1986).

20 Kong, J. & Yu, S. Fourier transform infrared spectroscopic analysis of protein secondary structures. *Acta Biochim Biophys Sin (Shanghai)* **39**, 549-559, doi:10.1111/j.1745-7270.2007.00320.x (2007).

21 Rahaman, H. *et al.* Heterogeneity of equilibrium molten globule state of cytochrome c induced by weak salt denaturants under physiological condition. *PLoS One* **10**, e0120465, doi:10.1371/journal.pone.0120465PONE-D-14-42826 [pii] (2015).

22 Mahato, M. *et al.* Hemoglobin-silver interaction and bioconjugate formation: a spectroscopic study. *J Phys Chem B* **114**, 7062-7070, doi:10.1021/jp100188s (2010).

23 Du, K. *et al.* Interaction of Ionic Liquid [bmin][CF3SO3] with Lysozyme Investigated by Two-Dimensional Fourier Transform Infrared Spectroscopy. *ACS Sustainable Chemistry & Engineering* **2**, 1420-1428, doi:10.1021/sc500218e (2014).

24 Lorber, B., Fischer, F., Bailly, M., Roy, H. & Kern, D. Protein analysis by dynamic light scattering: methods and techniques for students. *Biochem Mol Biol Educ* **40**, 372-382, doi:10.1002/bmb.20644 (2012).

25 Gunton, J. D., Shiryayev, A., Pagan, D.L. . Protein condensa-tion. Kinetic pathways to crystallization and disease. *Cambridge University Press*, 1–364 (2007).
